# Periodontitis in established rheumatoid arthritis patients: a cross-sectional clinical, microbiological and serological study

**DOI:** 10.1186/ar4061

**Published:** 2012-10-17

**Authors:** Menke de Smit, Johanna Westra, Arjan Vissink, Berber Doornbos-van der Meer, Elisabeth Brouwer, Arie Jan van Winkelhoff

**Affiliations:** 1 Center for Dentistry and Oral Hygiene, University of Groningen, University Medical Center Groningen, P.O. Box 30.001, 9700 RB Groningen, The Netherlands; 2 Department of Rheumatology and Clinical Immunology, University of Groningen, University Medical Center Groningen, P.O. Box 30.001, 9700 RB Groningen, The Netherlands; 3 Department of Oral and Maxillofacial Surgery, University of Groningen, University Medical Center Groningen, P.O. Box 30.001, 9700 RB Groningen, The Netherlands; 4 Department of Medical Microbiology, University of Groningen, University Medical Center Groningen, P.O. Box 30.001, 9700 RB Groningen, The Netherlands

## Abstract

**Introduction:**

The association between rheumatoid arthritis (RA) and periodontitis is suggested to be linked to the periodontal pathogen *Porphyromonas gingivalis*. Colonization of *P. gingivalis *in the oral cavity of RA patients has been scarcely considered. To further explore whether the association between periodontitis and RA is dependent on *P. gingivalis*, we compared host immune responses in RA patients with and without periodontitis in relation to presence of cultivable *P. gingivalis *in subgingival plaque.

**Methods:**

In 95 RA patients, the periodontal condition was examined using the Dutch Periodontal Screening Index for treatment needs. Subgingival plaque samples were tested for presence of *P. gingivalis *by anaerobic culture technique. IgA, IgG and IgM antibody titers to *P. gingivalis *were measured by ELISA. Serum and subgingival plaque measures were compared to a matched control group of non-RA subjects.

**Results:**

A higher prevalence of severe periodontitis was observed in RA patients in comparison to matched non-RA controls (27% versus 12%, p < 0.001). RA patients with severe periodontitis had higher DAS28 scores than RA patients with no or moderate periodontitis (p < 0.001), while no differences were seen in IgM-RF or ACPA reactivity. Furthermore, RA patients with severe periodontitis had higher IgG- and IgM-anti *P. gingivalis *titers than non-RA controls with severe periodontitis (p < 0.01 resp. p < 0.05), although subgingival occurrence of *P. gingivalis *was not different.

**Conclusions:**

Severity of periodontitis is related to severity of RA. RA patients with severe periodontitis have a more robust antibody response against *P. gingivalis *than non-RA controls, but not all RA patients have cultivable *P. gingivalis*.

## Introduction

Several studies have demonstrated an increased prevalence of periodontitis and a higher rate of tooth loss in patients with rheumatoid arthritis (RA) in comparison with the general population in the US [1,2], Northern Europe [3-6], and Australia [7]. RA may also be more prevalent among patients with periodontitis [4,8]. Differences in disease criteria and methods for evaluation of periodontal status, however, form a problem in interpretation of the literature [9,10].

Periodontitis and RA are both chronic destructive inflammatory disorders and result from deregulation of the host inflammatory response. Both conditions are potentiated by an exaggerated inflammatory response featuring an increase in local and perhaps circulating pro-inflammatory mediators, resulting in destruction of the soft and hard tissues surrounding the teeth (the periodontium) and the synovial joint [11-14]. Susceptibility is influenced by shared genetic and lifestyle factors. Both diseases are cumulative; that is, severity and loss of function increase with longer disease duration.

There are a number of postulated mechanisms by which infections can trigger autoimmune disease, but most evidence in animal models supports the idea that cross-reactive immune responses cause autoimmunity because of molecular mimicry between microbiological and self-antigens [15]. Rosenstein and colleagues [16] have hypothesized that *Porphyromonas gingivalis*, a major periodontal pathogen, plays a role in the pathogenesis of RA. *P. gingivalis *is a prokaryote that uniquely contains a peptidyl arginine deiminase enzyme [17] necessary for citrullination and can induce an immune response to citrullinated self-proteins [16,18]. Citrullination changes the structure and function of proteins and has been demonstrated in several physiological and pathological processes [19]. Antibodies against citrullinated proteins (ACPAs) are 95% specific and 68% sensitive for RA [20,21]. These antibodies can be present several years before the clinical onset of RA [22] and are associated with a more destructive disease course than RA without detectable ACPAs [23]. Moreover, periodontitis can contribute to the total inflammatory burden by eliciting bacteremia and systemic inflammatory responses [24,25]. Given the observed epidemiological association and the hypotheses mentioned above, periodontitis may be regarded as a risk factor for initiation and progression of RA. At present, disease management of RA is based on early diagnosis, aggressive treatment, and regular monitoring, and disease remission is the ultimate treatment goal. To achieve this goal, reduction of total inflammatory burden is necessary. This may involve periodontal infection control in patients with periodontitis.

Studies have reported higher antibody titers against *P. gingivalis *in RA patients and a positive correlation with ACPAs [26-28], suggesting that infection with this periodontal pathogen may play a role in the risk and progression of RA. However, oral colonization by *P. gingivalis *in patients with RA is barely considered. To study whether the association between periodontitis and RA is dependent on *P. gingivalis*, we compared host immune responses in RA patients with or without periodontitis in relation to the presence of cultivable *P. gingivalis *from subgingival plaque. Because of the recent observation that the inflamed periodontium contains citrullinated proteins [29], we also investigated the presence of ACPAs in the inflammatory exudates from the gingival crevice (gingival crevicular fluid, or GCF).

## Materials and methods

### Patients

#### Patients with rheumatoid arthritis

Established RA patients matching the inclusion criteria were consecutively recruited between March and September 2011 at the outpatient clinic of the Rheumatology and Clinical Immunology Department of the University Medical Center Groningen in Groningen. The inclusion criterion was fulfilling the American College of Rheumatology classification criteria for RA [30], and exclusion criteria were age under 18 years, edentulism, diabetes, active thyroid disease, presence of non-oral infections, present malignancy, myocardial infarction or stroke fewer than 6 months prior to the study, pregnancy including a 6-month post-partum period as well as breastfeeding and antibiotic use fewer than 3 months prior to the study. Assuming that the prevalence of periodontitis is 10% to 15% [31] and the odds ratio of periodontitis in RA is 1.8 to 2.0 [1,7], we calculated that we needed a minimum sample size of 75 patients with RA to find a difference of 12.5% in the prevalence of periodontitis by using a two-sided binomial test.

#### Non-rheumatoid arthritis controls

As a reference group for microbiological and serological measurements, subjects without RA were recruited from among subjects planned for first consultation at the Department of Dentistry and Oral Hygiene of the University Medical Center Groningen. The procedures of recruitment and informed consent were the same as for patients with RA. The inclusion criterion was not having RA, and exclusion criteria were the same as mentioned for patients with RA. Non-RA controls were matched for age, gender, number of teeth, body mass index (BMI), and smoking and periodontal status. We aimed to include matched non-RA controls in a ratio of 2:1 (Table 1). Healthy controls were defined as subjects without periodontitis and without cultivable subgingival *P. gingivalis*.

**Table 1:** Characteristics of patients with rheumatoid arthritis (RA), non-RA controls, and healthy controls

	Patients with RA	Non-RA controls	Healthy controls
Number	95	44	36

Female, percentage	68	57	56

Age in years (SD)	56 (11)	54 (9.7)	34 (15)

Current smoker, percentage	23	27	14

Former smoker, percentage	40	43	0

Body mass index	25.8 (4.9)	25.4 (4.4)	NA

Number of teeth	23.4 (6.3)	24.2 (4.9)	27.8 (2.9)

RA duration in years (SD)	7.4 (5.9)	0	0

DAS28 (SD)	2.4 (0.93)	0	0

Seropositive for IgM-RF, percentage	53	0	NA

Seropositive for ACPAs, percentage	71	NA	NA

Medication for RA, percentage			

None	6	100	100

DMARDs	79	0	0

Anti-TNFα	15	0	0

ACPA, anti-citrullinated protein antibody; anti-TNFα, anti-tumor necrosis factor-alpha; DAS28, disease activity score 28 tender and swollen joint count; DMARD, disease-modifying anti-rheumatic drug; IgM-RF, immunoglobulin M-rheumatoid factor; NA, not assessed; SD, standard deviation.

#### Control cohort for estimating prevalence of periodontitis in the general population

The prevalence of periodontitis was assessed in subjects attending a general dental practice within the referral area (Clinic for General Dental Practice Solwerd in Appingedam, The Netherlands). This control population consisted of 420 age- and gender-matched consecutive patients in whom the Dutch periodontal screening index (DPSI) score was assessed during one year (2010). The DPSI is a validated index based on bleeding on probing, pocket probing depth, and clinical attachment loss [32]. On the basis of DPSI scores, patients were categorized as having no periodontitis (A), moderate periodontitis (B), or severe periodontitis (C). Assessment of the socioeconomic status of this group was made according to data of highest self-reported level of received education of the municipal public health service of the northeast region of Groningen [33].

### Ethics approval

All participants provided written informed consent prior to study enrollment according to the Declaration of Helsinki (General Assembly October 2008), and this study was conducted with the approval of the Medical Ethical Committee of the University Medical Center Groningen (METc UMCG 2011/010).

### Clinical examination of rheumatoid arthritis

RA disease activity was measured with the Disease Activity Score 28 joint count (DAS28) [34]. Other parameters were disease duration of RA, smoking status (current, former, or never), BMI, and RA medication, including steroids and anti-tumor necrosis factor biologic agents. Assessment of the socioeconomic status was made according to highest self-reported level of received education.

### Clinical examination of periodontitis

Periodontal condition was examined by using DPSI. The DPSI score was taken by a periodontist blinded for the diagnosis of RA. In the general dental practice, the DPSI score was taken by the dentist at the first visit of the patient.

### Sampling

Peripheral blood and subgingival samples were obtained from the RA and non-RA controls at the day of clinical examination. Subgingival samples were taken by using sterile paper points [35]. Microbiological sampling included selection of the deepest bleeding periodontal pocket in each quadrant of the dentition on the basis of pocket probing depth measurements. If there were no bleeding periodontal pockets, mesial sites of the first molars or, in absence of a first molar, the mesial site from the adjacent anterior tooth in the dental arch was selected. Sample sites were isolated with cotton rolls, and supragingival plaque was carefully removed with curettes and cotton pallets. Subsequently, two paper points were inserted to the depth of the pocket and left in place for 10 seconds. All paper points per subject were pooled in reduced transport fluid [36] and processed for microbiological examination immediately after sampling. GCF samples were collected in the same way. The deepest non-bleeding site per quadrant was used to collect GCF to avoid blood contamination of the sample. GCF samples were discarded for further analysis if they were visibly contaminated with blood. Paper points for the GCF sample were pooled per subject in phosphate-buffered saline (PBS) with 1% bovine serum albumin. The paper points were removed after centrifuging at 28,000 *g *for 10 minutes, and the supernatant was stored at -20°C until use.

### Laboratory procedures

Serum was investigated for C-reactive protein (CRP) concentration by enzyme-linked immunosorbent assay (ELISA) (DuoSet; R&D Systems, Abingdon, UK). IgM-RF (in international units per milliliter) was measured by using an in-house validated ELISA (cutoff point for positivity: 25 U/mL) [37]. Total IgG anti-cyclic citrullinated protein (anti-CCP) antibodies (ACPAs) (in units per milliliter) were determined by using the Phadia analyzer (Phadia Laboratory Systems, Phadia AB, Uppsala, Sweden) with an upper detection limit of 340 U/mL (cutoff point for positivity: 10 U/mL). Antibody levels to five synthetic native peptides representing the best-established auto-antigens in RA [38] (that is, enolase-1 fibrinogen-1, fibrinogen-2, fibrinogen-3, and vimentin-1 and their citrullinated forms) were measured by using the same ELISA as described by van de Stadt and colleagues [39].

IgA, IgG, and IgM antibodies to *P. gingivalis *were determined by using an in-house developed ELISA, in which a pooled lysate of four randomly selected clinical isolates of *P. gingivalis *from patients with periodontitis was used as an antigen. These monocultures were suspended in ice-cold PBS with protease inhibitors (Complete Mini EDTA-free Protease Inhibitor Cocktail Tablets; Roche Diagnostics, Basel, Switzerland). After centrifuging and discarding of the supernatant, pellets were resuspended in an ice-cold lysis buffer containing non-denaturing detergent (Noninet P-40; Sigma-Aldrich, St. Louis, MO, USA) and sonicated for 15 minutes (Bioruptor Standard sonication device; Diagenode s.a., Liège, Belgium). Protein concentration was determined by using the BCA Protein Assay Kit (Pierce Protein Biology Products, Thermo Fisher Scientific, Rockford, IL, USA). Microtiter plates (Costar 96-Well; Corning, Amsterdam, The Netherlands) were coated overnight with 1 μg/mL of antigen. Standard curves were made from protein standard (N protein-standard SL; Siemens Healthcare Diagnostics, Den Haag, The Netherlands) diluted twofold (highest standard curve values were for 300, 25, and 250 ng/mL for IgA, IgG, and IgM, respectively). For the standard curves, the following capture antibodies were used: monoclonal anti-human IgA (1:4,000, clone GA-112; Sigma-Aldrich, Zwijndrecht, The Netherlands), monoclonal anti-human IgM (1:10,000, clone MB-11, μ-chain-specific; Sigma-Aldrich), and goat anti-human IgG (1:5,000, F(ab')_2 _fragment-specific; Jackson ImmunoResearch Europe Ltd., Newmarket, UK), respectively. Serum samples were incubated in fourfold serial dilutions (1:100, 1:400, 1:1,600, and 1:6,400). Detection of anti-*P. gingivalis *antibodies was carried out with horseradish peroxidase-labeled goat anti-human IgA, mouse anti-human IgG (Fc fragment-specific, clone JDC-10), and mouse anti-human IgM (μ-chain-specific, clone SA-DA4; all from SouthernBiotech, Birmingham, AL, USA) followed by color reaction with tetramythylbenzidin and hydrogen peroxide. Absorbance was read at 450 nm in an EMax microplate reader and corrected for background binding, and concentration of antibodies was calculated by SOFTmax PRO software (Molecular Devices, Sunnyvale, CA, USA) according to the IgA, IgG, or IgM standard curves included on each ELISA plate and expressed in nanograms per milliliter. As an internal control, two serum samples with a repeatedly high and a low titer were tested at each plate. The variation coefficients were 22% for IgA, 20% for IgG, and 25% for IgM anti-*P. gingivalis*. Interference of IgM-RF was investigated by spiking samples with sera with known high titers of RF. Adding RF had no measurable effect on anti-*P. gingivalis *titers.

In paired samples of serum and GCF of patients with RA, the presence of IgG-ACPAs was assessed by using the anti-CCP2 ELISA (Euro Diagnostica, Nijmegen, The Netherlands). Because the HLA-DRB1 shared epitope (SE) is a known genetic risk factor for RA and a possible genetic risk factor for periodontitis [40], HLA-DRB1-SE-containing alleles were genotyped from genomic DNA in the patients with RA. The presence of an RA SE was analyzed by a polymerase chain reaction-based sequence-specific oligonucleotide probe hybridization (SSOP) approach by using a commercial kit (Hologic Gen-Probe Incorporated, San Diego, CA, USA) and Luminex xMAP technology (Luminex Corporation, Austin, TX, USA) in accordance with the instructions of the manufacturer.

### Microbiology

Microbiological samples were processed by using standard anaerobic culture techniques [41,42]. Paper point samples were vortexed for 2 minutes, and appropriate 10-fold serial dilutions (100 μL) in PBS were plated on blood agar plates (Oxoid no. 2; Oxoid Limited, Basingstoke, UK), which were supplemented with horse blood (5% vol/vol), hemin (5 mg/L), and menadione (1 mg/L) and incubated in 80% N_2_, 10% H_2_, and 10% CO_2 _at 37°C for up to 14 days. Besides the presence and proportions of *P. gingivalis*, those of other established periodontal pathogens, including *Prevotella intermedia*, *Fusobacterium nucleatum*, *Parvimonas micra*, *Tannerella forsythia*, and *Campylobacter rectus*, were assessed [43]. Identification was based on microscopic morphology, Gram reaction, and the production of a set of metabolic enzymes (API/ID 32; BioMérieux, La Balme Les Grottes, France). Additional tests for identification included detection of a trypsin-like activity based on the degradation of benzoyl-DL-arginine-2-naphthylamide (Sigma-Aldrich) [44]. Finally, the total number of colony-forming units per sample was determined.

### Statistical analysis

Data were analyzed by using GraphPad Prism 4 and Instat 3 (GraphPad Software, Inc., San Diego, CA, USA). For group comparisons among two groups, the unpaired two-tailed *t *test for variables with normal distribution and the two-tailed Mann-Whitney test for skewed variables were used. For group comparisons among three groups, Kruskal-Wallis one-way analysis of variance was performed. The Fisher exact test or the chi-squared test for independence was used to analyze contingency tables. Significance level α was 0.05.

## Results

### Patients

Two hundred thirty-nine consecutive patients with RA were invited to participate in this study. Of the 167 patients who provided written informed consent, 66 patients met at least one of the exclusion criteria. Of the 101 included patients, six patients were excluded from analysis because of incomplete data. The final cohort consisted of 95 patients with RA. In addition, 203 consecutive patients were invited to join the control group. Of this group, 108 dentate persons without RA provided written informed consent. Twenty-eight of them had to be expelled on the basis of the exclusion criteria. The remaining 80 patients served as non-RA controls for blood and subgingival samples, matched for age, gender, BMI, and periodontal and smoking status (*n *= 44), or as healthy controls (no periodontitis and no cultivable *P. gingivalis*, *n *= 36). Characteristics of the predominantly (99%) Caucasian patients with RA, non-RA controls, and the healthy controls are summarized in Table 1. The general control population for estimating the prevalence of periodontitis consisted of 420 individuals matched for age and gender with the patients with RA (age of 56 ± 5.5 years, and 68% were female). The socioeconomic status of this group as assessed by the highest self-reported level of received education was comparable between the control population and the patients with RA (low: 27% versus 36%, middle: 37% versus 30%, and high: 36% versus 34%).

### Periodontitis

Forty-three percent of the patients with RA had moderate periodontitis, and 27% had severe periodontitis. These numbers are significantly higher than, respectively, the 18% and 12% found in the control population (*P *< 0.001). Compared with the control population, the relative risk for having RA and severe periodontitis was 3.7 (95% confidence interval of 2.4 to 5.9) (Table 2).

**Table 2:** Prevalence of periodontitis of rheumatoid arthritis patients compared with the age- and gender-matched control population

DPSI category	Patients with RA (*n *= 95)	Control population (*n *= 420)	*P *value	Relative risk	95% CI
A: No periodontitis, percentage	30	70	< 0.001		

B: Moderate periodontitis, percentage	43	18	< 0.001	3.6	2.3-5.5

C: Severe periodontitis, percentage	27	12	< 0.001	3.7	2.4-5.9

CI, confidence interval; DPSI, Dutch periodontal screening index; RA, rheumatoid arthritis.

**Figure 1:**
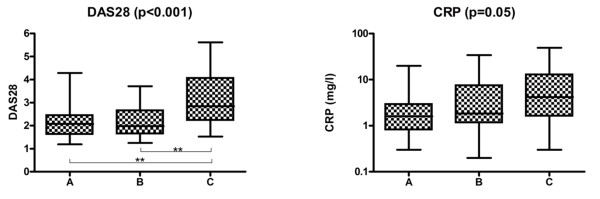
**DAS28 scores and CRP levels in patients with rheumatoid arthritis and no (a), moderate (b), or severe (c) periodontitis**. **P < 0.01. CRP, C-reactive protein; DAS28, disease activity score 28 tender and swollen joint count.

RA patients with severe periodontitis had significantly higher DAS28 scores (*P *< 0.001) than RA patients with no or moderate periodontitis (Figure 1). CRP levels in RA patients with severe periodontitis were higher than in RA patients without periodontitis (marginally significant, *P *= 0.05). In none of the DPSI categories was there a difference in DAS28 scores between smokers and non-smokers or former smokers. Also, no difference was found in RA disease duration, although patients with severe periodontitis were significantly older (*P *< 0.01). In the control population, there were no age differences between the DPSI categories.

### Presence of HLA-DRB1 shared epitope in patients with rheumatoid arthritis

No differences were found in the presence or absence of the HLA-DRB1-SE in RA patients with no, moderate, or severe periodontitis (*n *= 78). In RA patients with no, moderate, or severe periodontitis, 58%, 66%, and 62%, respectively, were positive for HLA-DRB1-SE, and 29%, 21%, and 15%, respectively, had two alleles.

### Serology

**Figure 2:**
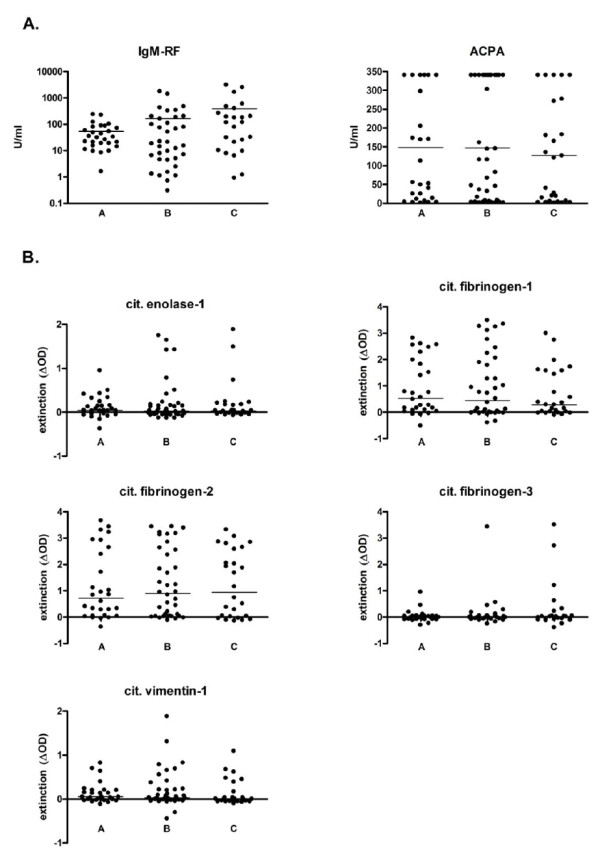
**(A) IgM-RF and ACPA reactivity and (B) reactivity against five citrullinated (cit.) peptides in patients with rheumatoid arthritis and no (a), moderate (b), or severe (c) periodontitis**. No significant differences were observed. ΔOD, optical density of the citrullinated form minus the native form of the peptide; ACPA, anti-citrullinated protein antibody; IgM-RF, immunoglobulin M-rheumatoid factor.

Between RA patients with no, moderate, or severe periodontitis, no differences were seen in IgM-RF and ACPA levels or in reactivity against the citrullinated peptides enolase-1, fibrinogen-1, -2, and -3, and vimentin-1 (Figure 2). Between RA patients culture-positive or -negative for *P. gingivalis*, only reactivity against citrullinated fibrinogen-2 was different; reactivity was higher in *P. gingivalis*-positive patients (*P *< 0.01). A small number of RA patients with moderate (*n *= 2) or severe (*n *= 2) periodontitis and culture-positive for *P. gingivalis *had a higher reactivity against citrullinated α-enolase compared with RA patients without periodontitis and without subgingival *P. gingivalis*. This *P. gingivalis *culture-positive subgroup also had high IgM-RF (mean 539 ± 796 U/mL), ACPAs (mean 340 ± 0 U/mL), and anti-*P. gingivalis *titers (IgM: mean 93 ± 135, IgG: 190 ± 357, IgA: 61 ± 81 mg/L).

**Figure 3:**
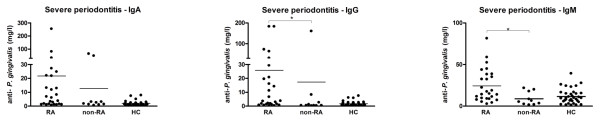
**IgA, IgG, and IgM antibody response against *Porphyromonas gingivalis *in rheumatoid arthritis (RA) patients and non-RA controls with severe periodontitis as well as in healthy controls (HC)**. *P < 0.05. Ig, immunoglobulin.

Overall, patients with RA showed higher IgM anti-*P. gingivalis *titers compared with non-RA controls (*P *< 0.05). There were no differences in anti-*P. gingivalis *titers between RA patients and non-RA controls with no or moderate periodontitis; however, RA patients with severe periodontitis showed both higher IgG and IgM anti-*P. gingivalis *titers compared with non-RA controls with severe periodontitis (*P *< 0.05) (Figure 3). RA patients with moderate periodontitis have a less pronounced anti-*P. gingivalis *response compared with RA patients with severe periodontitis but a higher one than non-RA controls with severe periodontitis for IgG (borderline significant, *P *= 0.06) and IgM (*P *< 0.05). Both RA patients and non-RA controls culture-positive for *P. gingivalis *showed higher anti-*P. gingivalis *titers compared with their culture-negative counterparts.

In patients with RA, serum levels of IgM-RF and ACPAs showed a strong correlation (ρ = 0.51, *P *< 0.0001), as did IgG anti-*P. gingivalis *with IgM anti-*P. gingivalis *(ρ = 0.41, *P *< 0.0001) and IgA anti-*P. gingivalis *(ρ = 0.66, *P *< 0.0001). Medication used for RA was not of influence on *P. gingivalis *titers. In non-RA controls, IgG anti-*P. gingivalis *only correlated with IgA anti-*P. gingivalis *(ρ = 0.65, *P *< 0.0001).

**Figure 4:**
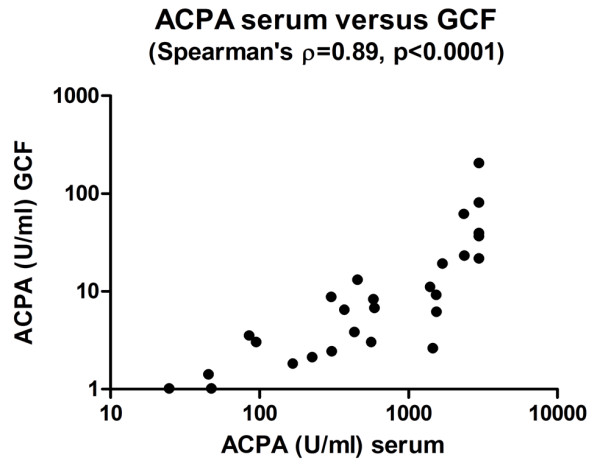
**Correlation of anti-citrullinated protein antibody (ACPA) in paired samples of serum and gingival crevicular fluid (GCF) of patients with rheumatoid arthritis (*n *= 45)**.

In patients with RA, there was a weak but significant correlation between IgM anti-*P. gingivalis *and IgM-RF (ρ = 0.33, *P *< 0.01), ACPAs (ρ = 0.24, *P *< 0.05), and the citrullinated peptides fibrinogen-1 (ρ = 0.27, *P *< 0.05) and fibrinogen-3 (ρ = 0.22, *P *< 0.05). Likewise, IgG anti-*P. gingivalis *was correlated with IgM-RF (ρ = 0.26, *P *< 0.05). In RA patients with severe periodontitis, there were no correlations between IgG, IgM, and IgA anti-*P. gingivalis *titers and IgM-RF and ACPAs. In patients with RA, ACPA levels in serum were comparable to ACPA levels in paired GCF samples which were not visibly contaminated with blood (*n *= 45, ρ = 0.89, *P *< 0.0001) (Figure 4).

### Microbiology

The subgingival prevalence of *P. gingivalis *was not different between patients with RA (16%) and non-RA controls (20%). In both groups, *P. gingivalis *was infrequently cultured in the absence of periodontitis (6% to 12%). None of the other identified periodontal pathogens differed in prevalence between RA patients and non-RA controls (*P. intermedia*: 66% versus 73%, *T. forsythia*: 84% versus 70%, *P. micra*: 88% versus 89%, *F. nucleatum*: 100% versus 95%, and *C. rectus*: 33% versus 41%). However, the prevalence of anaerobic Gram-negative rods other than *P. gingivalis *and *P. intermedia *was higher in patients with RA (27% versus 8%, *P *< 0.05), as was the total bacterial load (in colony-forming units per milliliter) (*P *< 0.05), notwithstanding the comparable mean probing pocket depth between RA patients and matched non-RA controls (4.2 ± 1.0 mm).

## Discussion

In this study, we found a significantly increased prevalence of periodontitis in RA patients and the highest RA disease activity in patients with severe periodontitis. Importantly, serological markers for systemic inflammation of periodontal origin further substantiated the connection of the two diseases in terms of higher antibody titers against *P. gingivalis *in RA patients with severe periodontitis compared with severe periodontitis patients without RA. These differences cannot be explained by differences in *P. gingivalis *colonization, since the distributions in RA and non-RA patients were similar.

Given that periodontitis is associated with only moderate elevations of CRP and erythrocyte sedimentation rate levels [45,46], the higher DAS28 scores observed in RA patients with periodontitis can only partly be explained by these higher levels due to periodontitis. This is the first study that focused on anti-*P. gingivalis *titers in RA and non-RA controls, equally distributed in prevalence of *P. gingivalis *and periodontitis. We confirm elevated antibody titers against *P. gingivalis *in RA patients as reported in former studies; however, these studies did not consider the periodontal status or the microbiology of the patients with RA [27,28]. We found higher anti-*P. gingivalis *titers in RA patients compared with matched non-RA controls in cases of severe periodontitis. No differences in anti-*P. gingivalis *titers between these groups were found in cases of moderate periodontitis; however, RA patients with moderate periodontitis still had higher titers compared with severe periodontitis patients without RA. RA patients with cultivable *P. gingivalis *showed the highest anti-*P. gingivalis *titers, suggesting that RA amplifies the antibody response against *P. gingivalis *and that colonization of *P. gingivalis *could have contributed to the elevated titer, according to the fact that carriage of *P. gingivalis *is the strongest determinant of the systemic antibody response against this periodontal pathogen [47]. An explanation for the elevated anti-*P. gingivalis *titers could be a hyperinflammatory state of RA patients with periodontitis. ACPAs directed to citrullinated peptides of *P. gingivalis *could be another explanation for the elevated titers. Lundberg and colleagues [48] showed cross-reactivity of human ACPAs with bacterial enolase. We found a high reactivity in a small subgroup of RA patients with periodontitis and culture-positive for *P. gingivalis*, but overall we found a weak correlation between ACPAs and anti*-P. gingivalis *titers. Whether anti-*P. gingivalis *antibodies are directed against the same epitopes as ACPAs needs further investigation. To consider whether elevated titers are specific for *P. gingivalis*, antibody titers to other periodontal bacteria should be assessed.

Little is known about oral colonization by *P. gingivalis *in patients with RA. We found no differences in the presence and proportions of any of the assessed periodontal pathogens between RA patients and non-RA controls; however, the prevalence of anaerobic Gram-negative rods other than *P. gingivalis *and *P. intermedia *was higher in patients with RA. Recently, Scher and colleagues [49] assessed the complete subgingival microbiota by pyrosequencing and detected *P. gingivalis *more frequently in recently diagnosed, never-treated RA patients (*n *= 25) in comparison with established RA patients or healthy controls; this finding, however, could be explained by the higher prevalence of severe periodontitis in those patients.

An association between the presence of periodontitis and ACPA or IgM-RF levels or both could not be established, in contrast to the findings of Dissick and colleagues [2], nor could we find differences in reactivity to the five synthetic citrullinated peptides between the DPSI categories, other than a higher reactivity against citrullinated peptide fibrinogen-2 in *P. gingivalis *culture-positive patients. As epitope spreading is related to RA disease duration [39], a widespread reactivity to different citrullinated peptides was found in our cohort with a relatively long disease duration. In future studies, the (temporal) relation of RA, periodontitis, and antibodies specific for RA should be performed in periodontitis patients, in non-treated RA patients, and/or in patients at risk for RA, ideally in a prospective follow-up study.

We found no differences in the presence or absence of the HLA-DRB1-SE in RA patients with no, moderate, or severe periodontitis, although this should be interpreted with some caution because of the small numbers of patients. The strong correlation between ACPA levels in serum and GCF, with lower levels in GCF, is suggestive of diffusion of ACPAs from plasma to GCF. Within the limitations of the method used in this study, we found no indication for local ACPA production in the periodontium.

## Conclusions

We confirmed the previously reported disease association between RA and periodontitis and the increased prevalence of periodontitis in patients with RA. In addition, the severity of periodontitis appeared to be related to RA disease activity. Furthermore, severity of RA was not associated with cultivable *P. gingivalis *in established RA patients, although anti-*P. gingivalis *titers were higher in RA patients with severe periodontitis compared with matched non-RA subjects.

## Abbreviations

ACPA: anti-citrullinated protein antibody; anti-CCP: anti-cyclic citrullinated protein; BMI: body mass index; CRP: C-reactive protein; DAS28: disease activity score 28 tender and swollen joint count; DPSI: Dutch periodontal screening index; ELISA: enzyme-linked immunosorbent assay; GCF: gingival crevicular fluid; PBS: phosphate-buffered saline; RA: rheumatoid arthritis; RF: rheumatoid factor; SE: shared epitope.

## Competing interests

The authors declare that they have no competing interests.

## Authors' contributions

All authors were involved in drafting the article or revising it critically for important intellectual content, and all authors approved the final version to be published. All authors were involved in study conception and design. Acquisition and analysis of data were carried out by MdS, JW, EB, and BDvdM, who take responsibility for the integrity of the data and the accuracy of the data analysis.
